# Targeted Therapy for Idiopathic Inflammatory Myopathy

**DOI:** 10.1002/jcsm.70143

**Published:** 2025-12-10

**Authors:** Ruijie Wang, Wei Lin, Qibing Xie, Geng Yin

**Affiliations:** ^1^ Department of Rheumatology and Immunology West China Hospital, Sichuan University Chengdu Sichuan China; ^2^ Department of General Practice, General Practice Medical Center West China Hospital, Sichuan University Chengdu Sichuan China

**Keywords:** biological products, CAR‐T, idiopathic inflammatory myopathies, immunotherapy, molecular targeted therapy, myositis

## Abstract

**Background:**

Idiopathic inflammatory myopathies (IIM) are a heterogeneous group of autoimmune disorders characterized by chronic muscle inflammation and significant extramuscular involvement. A substantial proportion of patients exhibit refractory or relapsing disease despite conventional immunosuppressive therapies, necessitating the development of novel targeted treatments. Recent immunological advances have identified novel therapeutic targets—including B cells, T cells, cytokines and intracellular signalling pathways—paving the way for personalized treatment. Targeted therapies represent promising new approaches.

**Methods:**

This comprehensive review synthesizes current evidence on targeted therapies for IIM. We systematically searched PubMed, the Cochrane Library and Google Scholar for studies published before September 2025, including randomized controlled trials, retrospective and observational studies, meta‐analyses and case reports. We synthesized evidence on the efficacy and safety of various targeted treatments across IIM subtypes and complications, highlighting recent advances and future directions.

**Results:**

Targeted therapies are revolutionizing IIM management. B‐cell‐targeted therapies constitute a relatively established modality. Rituximab has become a cornerstone therapy for refractory disease, as supported by high‐level evidence. Modulation of T‐cell costimulation with abatacept benefits specific subtypes. JAK inhibitors show remarkable efficacy, particularly for cutaneous manifestations and interstitial lung disease. Cytokine inhibitors, proteasome inhibitors and low‐dose IL‐2 also show benefits across various IIM subtypes and complications. Novel mechanisms are emerging, including Fc receptor antagonism (efgartigimod) to reduce pathogenic IgG and advanced cellular therapies such as CAR‐T‐cell therapy and bispecific T‐cell engagers (BiTEs), which have induced sustained remission in severe refractory cases. The heterogeneous treatment responses observed between and within IIM subtypes present a central challenge, largely stemming from their diverse underlying immunopathogenic mechanisms. The primary safety concern remains infection risk, necessitating individualized benefit–risk assessment.

**Conclusion:**

The targeted therapy landscape in IIM is rapidly expanding, enabling more precise and effective management. These strategies show significant promise in improving outcomes for patients with refractory disease. Future efforts should focus on optimizing treatment selection through biomarker discovery, conducting larger randomized trials to strengthen the evidence base, and managing long‐term safety.

## 1 Introduction

Idiopathic inflammatory myopathies (IIM), commonly referred to as myositis, are a heterogeneous group of autoimmune diseases characterized by chronic muscle inflammation and progressive muscle weakness. The incidence of IIM is estimated to range from 0.2 to 2 cases per 100 000 person‐years, with a prevalence of 2–25 cases per 100 000 person‐years [1]. The clinical manifestations of IIM are diverse, ranging from muscle weakness to systemic involvement, such as interstitial lung disease (ILD) and skin lesions, which significantly impair patients' quality of life. On the basis of clinical, histological and serological features, IIM can be divided into dermatomyositis (DM), anti‐synthetase syndrome (ASS), immune‐mediated necrotizing myopathy (IMNM), inclusion body myositis (IBM), polymyositis (PM) and overlap myositis [2]. Despite advances in understanding the pathogenesis of IIM, the exact aetiology remains unclear.

Traditional management of IIM has relied on high‐dose glucocorticoids and immunosuppressants. While these therapies can relieve symptoms in many patients, a substantial subset continues to present with refractory or relapsing disease, with some even experiencing long‐term sequelae and significant functional impairments, which pose a major challenge in clinical management. These findings have laid the foundation for targeted therapies for IIM, offering the potential for more precise and effective treatment by targeting specific cells, molecules or signalling pathways.

**FIGURE 1:**
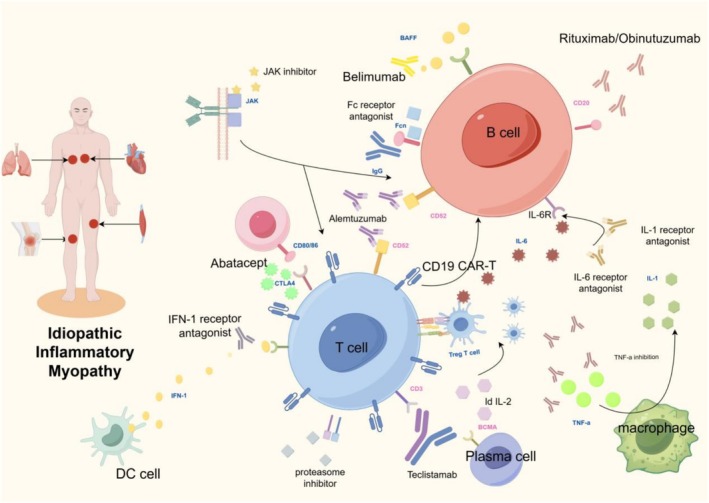
The targeted therapy mechanism map of idiopathic inflammatory myopathy.

Currently, targeted therapies for IIM include B‐cell‐targeted therapies, T‐cell‐targeted therapies, cytokine inhibitors, JAK inhibitors, proteasome inhibitors and emerging approaches such as CAR‐T‐cell therapy, BiTE and low‐dose IL‐2 therapy. The targeted therapies discussed in this article have been systematically categorized and summarized in Table 1, which provides a clear comparison of their respective mechanisms of action, clinical efficacy, safety profiles and levels of evidence, with corresponding references appropriately cited. These strategies have demonstrated promising results in clinical trials and real‐world studies. They aim to modulate specific immune pathways associated with IIM, with the hope of benefiting patients with refractory disease. This article contains a summary diagram summarizing the mechanisms and targets of targeted therapy for IIM (Figure 1). However, variable responses among different IIM subtypes, long‐term safety issues and the high cost of some therapies remain unresolved challenges.

**TABLE 1:** Summary of targeted therapies for IIM.

Therapeutic category	Drug name(s)	Mechanism of action	Key clinical findings and efficacy	Notable safety concerns	Level of evidence	References
B‐cell targeting	Rituximab	Depletes CD20+ B cells via ADCC/CDC	Effective in refractory IIM ^a^ Improved ILD ^b^ in IIM, particularly ASS Glucocorticoids‐sparing effect	Infusion reactions, infection, cytopenia and potential malignancy risk	High (RCT ^c^ s, meta‐analysis)	[ 3, 4, 5, 6, 7, 8, 9, 10, 11, 12, 13, 14, 15, 16 ]
	Obinutuzumab	Depletes CD20+ B cells with enhanced ADCC/CDC	Effective in treating rituximab‐refractory or hypersensitive IIM by improving respiratory and muscular symptoms	Well‐tolerated in reported cases	High (case series)	[ 17, 18, 19, 20 ]
	Belimumab	Inhibits B‐cell‐activating factor (BAFF)	Improved skin ulcers/calcinosis in retrospective studies No significant benefit with placebo in an RCT	No severe AEs reported in small studies	High (retrospective study, negative RCT)	[ 21, 22, 23, 24]
CAR‐T cell	CD19/BCMA CAR‐T	Targets and depletes B/plasma cells	Induced drug‐free remission in refractory IIM (ASS, ^d^ IMNM ^e^ and JDM ^f^ )	Cytopenia, CRS, infection, ICANS ^g^	Low (case series/reports)	[25, 26, 27, 28, 29, 30, 31, 32, 33]
BiTE	Teclistamab	Acts on T cells via CD3, targets plasmablasts and plasma cells through BCMA	Effective in refractory severe MDA5+ DM	Transient CRP ^h^ /cytokine elevation	Low (case report)	[34]
T‐cell targeting	Abatacept	Inhibits T‐cell co‐stimulation	~50% response in refractory PM ^i^ /DM Potential benefit in non‐DM subtypes and ASS‐ILD May improve JDM calcinosis	Infections, generally well‐tolerated	High (RCT, cohort study)	[ 35, 36, 37, 38, 39, 40, 41 ]
B and T cell targeting	Alemtuzumab	Depletes CD52+ B and T cells	Effective in refractory PM, by improving muscular symptoms Glucocorticoids‐sparing effect	Severe lymphopenia, infection risk	Low (case report)	[42]
TNF inhibitors	Etanercept	Acts as a soluble decoy receptor to neutralize TNF‐*α*	The efficacy is uncertain	Worsening myopathy with elevated CK, ^j^ rashes	High (RCT, case series)	[ 43, 44, 45 ]
	Infliximab	Binds and neutralizes TNF‐*α*.	Improved muscle and skin manifestations Effective against calcinosis Potential benefit with acute interstitial pneumonia	Infections, infusion reactions	High (RCT, cohort study and case reports)	[ 46, 47, 48, 49, 50 ]
	Adalimumab	Binds and neutralizes TNF‐*α*	Improved muscle and skin symptoms in JDM, potential benefit for ILD	Infections, potential for inducing autoimmune phenomena	Low (Cohort study, Case reports)	[ 47, 48, 49 ]
IFN‐I receptor antagonist	Anifrolumab	Blocks type I IFN signalling	Improved refractory cutaneous DM Rapid skin response Prednisone‐sparing effect	Infections (URTI and peritonsillar abscess)	Moderate (case series and retrospective study)	[ 51, 52, 53 ]
IL‐6 receptor antagonist	Tocilizumab	Targets the IL‐6 receptor	Beneficial in ASS and MDA5+ DM with RP‐ILD ^k^ Failed in general DM/PM RCT	Infections	High (mixed RCT and real‐world study)	[ 54, 55, 56, 57, 58, 59, 60 ]
IL‐1 receptor antagonist	Anakinra	Targets the IL‐1 receptor	Effective in myositis with extramuscular involvement Not effective in IBM	Well‐tolerated	Moderate (open‐label study and case reports)	[ 61, 62, 63, 64, 65, 66 ]
Fc receptor‐targeting	Efgartigimod	FcRn antagonist, reduces the level of IgG	Effective in refractory IMNM Reduced anti‐HMGCR/SRP antibodies	Mild headache	Moderate (preclinical + pilot clinical study)	[ 67, 68, 69 ]
JAK inhibitors	Tofacitinib	JAK1/JAK3 inhibitor	Improved skin symptoms in DM/ADM Improved ILD in MDA5+ DM/JDM	Increased virus infection risks, headache	Moderate (retrospective study and prospective study)	[70, 71, 72, 73, 74, 75]
	Baricitinib	JAK1/JAK2 inhibitor	Improved skin, muscle symptoms and ILD in DM/JDM	Herpes zoster, infections	High (RCT and case series)	[76, 77, 78, 79, 80]
	Upadacitinib	JAK1 inhibitor	Improved skin symptoms in refractory DM/ASS	Well‐tolerated	Low (case series)	[81, 82]
	Ruxolitinib	JAK1/JAK2 inhibitor	Improved skin symptoms in DM/JDM	Infections	Low (case reports)	[83, 84, 85, 86]
Proteasome inhibitor	Zetomipzomib	Targets the LMP7 and LMP2 subunits of the immunoproteasome	No significant efficacy advantage versus the control in RCT	Well‐tolerated	High (small RCT)	[87, 88]
Low‐dose IL‐2	Low‐dose IL‐2	Expands regulatory T cells (Tregs)	Reduced disease activity and improved skin symptoms in IIM Enhanced Treg numbers	Well‐tolerated	Moderate (prospective cohort)	[89, 90, 91, 92, 93, 94]

^a^ Idiopathic inflammatory myopathies.

^b^ Interstitial lung disease.

^c^ Randomized controlled trial.

^d^ Anti‐synthetase syndrome.

^e^ Immune‐mediated necrotizing myopathy.

^f^ Juvenile dermatomyositis.

^g^ immune effector cell‐associated neurotoxicity syndrome.

^h^ C‐reactive protein.

^i^ Polymyositis.

^j^ Creatine kinase.

^k^ Rapid‐progressive interstitial lung disease.

This review aims to provide a comprehensive overview of the latest advancements in targeted therapies for IIM, focusing on their mechanisms of action, clinical efficacy, safety profiles and future research directions. By summarizing the existing evidence and identifying research gaps, we hope to contribute to the ongoing quest for more effective and personalized treatment options for this challenging disease group.

## 2 B/Plasma Cell–Targeting Therapies

Rituximab (RTX) is a chimeric murine/human anti‐CD20 monoclonal antibody that depletes B cells through complement‐dependent cytotoxicity, antibody‐dependent cellular cytotoxicity and the induction of programmed cell death [3]. RTX reduces muscle‐infiltrating CD19+ B cells and CD68+ macrophages, thereby attenuating Type I IFN signatures and demonstrating therapeutic potential in refractory IIM.

In a randomized, double‐blind phase IIb trial, RTX and cyclophosphamide showed comparable efficacy in treating connective tissue diseases with ILD, including IIM, with fewer adverse events observed in the RTX group [4]. A prospective cohort study of patients with refractory active IIM revealed significant clinical improvement in PM but variable responses in DM. While some DM patients experience marked improvements in skin and muscle symptoms, others show no substantial changes in refractory skin lesions or muscle strength [5, 16]. In patients with severe refractory ASS, combination therapy with RTX and mycophenolate mofetil improved muscle enzyme levels, myocarditis and pulmonary function with a favourable safety profile [6]. Furthermore, RTX was effective in both anti‐synthetase antibody‐positive and antibody‐negative IIM, with a notable glucocorticoid‐sparing effect in anti‐synthetase‐positive patients [7]. Another study confirmed that RTX could reverse lung function decline in ASS‐associated ILD patients, with stable pulmonary function maintained during 2 years of maintenance therapy [8]. RTX has also shown efficacy in anti‐MDA5 antibody‐positive DM and juvenile DM (JDM) patients. After 12 months of RTX treatment, all patients discontinued glucocorticoids, with improvements observed in pulmonary, skin, and muscle symptoms [9].

With respect to treatment timing, RTX was found to be more effective in treatment‐naive IIM patients than in those with refractory disease, with early initiation associated with better outcomes [10]. In pregnant patients with IIM, RTX should be administered during early pregnancy to avoid the risk of neonatal B‐cell depletion and cytopenias [11]. In terms of dosage, studies have indicated that 1 g × 2 doses and 500 mg × 2 doses provide comparable efficacy, with both demonstrating glucocorticoid‐sparing effects, although the long‐term efficacy of low‐dose RTX requires further investigation [12]. A meta‐analysis of response rates to RTX reported a pooled complete response rate of 45% (*n* = 121), a partial response rate of 39% (*n* = 44) and an overall response rate of 65% (*n* = 480). The estimated incidence of adverse events was 18% (*n* = 135) [13].

Common adverse events associated with RTX include fever, rash, cytopenia, hypotension, back pain, arthralgia and myalgia [14]. Additionally, given the greater association between DM and malignancies, RTX treatment may delay cancer diagnosis, necessitating vigilance for potential risks [15].

Overall, RTX demonstrates significant efficacy and safety in the treatment of IIM and associated ILD, making it an ideal option for refractory IIM patients. RTX has been approved by NHS England for IIM and is recommended by the British Society for Rheumatology (BSR) and the American College of Rheumatology (ACR) for refractory IIM.

Obinutuzumab is a glycoengineered, humanized anti‐CD20 monoclonal antibody currently approved by the Food and Drug Administration(FDA) for the treatment of follicular lymphoma and chronic lymphocytic leukaemia. Compared with RTX, it exhibits enhanced complement‐dependent cytotoxicity and antibody‐dependent cellular cytotoxicity, along with reduced immunogenicity. This agent has recently been applied in the management of IIM. A case series described five patients with refractory IIM—two with ASS, one with DM, one with overlap myositis, and one with systemic lupus erythematosus (SLE)/DM overlap—four of whom had hypersensitivity to RTX and one who had previously failed RTX therapy. Following obinutuzumab treatment, respiratory symptoms improved, and pulmonary imaging and pulmonary function tests remained stable. In another case of refractory anti–Jo‐1 ASS, improvements  in myalgia, muscle strength, and creatine kinase levels were observed after one cycle of obinutuzumab. The aforementioned case reports demonstrated no significant adverse reactions, indicating the favourable safety profile of obinutuzumab. This agent shows considerable promise in treating patients with a suboptimal response to RTX [17, 19, 20].

Ocrelizumab, another humanized anti‐CD20 antibody, is FDA‐approved for multiple sclerosis. It was used in a refractory ASS patient without significant clinical benefit, but subsequent treatment with anti‐CD19 chimeric antigen receptor (CAR)‐T‐cell therapy led to improvement. Thus, the role of ocrelizumab in IIM remains to be further elucidated [25].

Daratumumab, a monoclonal antibody that targets CD38, exerts its effects by targeting not only B cells but also plasma cells. Emerging evidence suggests its potential efficacy in patients with severe clinical courses of anti‐MDA5 DM and anti‐SRP IMNM. Furthermore, daratumumab may represent a promising therapeutic option for IBM patients with anti‐cytosolic 5′‐nucleotidase 1A (cN1A) antibodies, warranting investigation in future studies [95, 96, 97, 98, 99].

B‐cell‐activating factor (BAFF) plays a critical role in B‐cell survival and maturation. Elevated serum BAFF levels are associated with various autoimmune diseases, including DM, PM, and IBM [21, 22]. Belimumab, a BAFF inhibitor, improves myositis symptoms by reducing serum BAFF levels. A retrospective study revealed that belimumab alleviates muscle pain and fatigue in refractory DM and JDM patients, with significant improvements in subcutaneous ulcers and calcinosis and no severe adverse events reported [23]. However, in a multicenter randomized controlled trial, no significant clinical differences were observed between the belimumab and placebo groups, indicating the need for further validation of the efficacy of belimumab in patients with IIM [24].

In summary, the safety profiles of B‐cell‐targeting agents require careful evaluation. RTX may induce infusion‐related reactions, increase the risk of infections and lead to adverse events, including cytopenias. Although preliminary data suggest a favourable safety profile for obinutuzumab, its potent B‐cell‐depleting activity could theoretically increase infection risk. By targeting plasma cells, daratumumab profoundly suppresses humoral immunity. Therefore, a comprehensive risk–benefit assessment, including screening for latent infections and verification of immunization status, is essential before initiating treatment.

## 3 CAR‐T‐Cell Therapy

CAR‐T‐cell therapy involves collecting T cells from individuals and genetically engineering them to express CARs that recognize target cells. After expansion, these CAR‐T cells are reinfused into patients, where they specifically target and proliferate to exert cytotoxic effects. Originally developed for the treatment of B‐cell malignancies, CAR‐T‐cell therapy has recently been explored for the treatment of autoimmune diseases because of the critical role of B cells in disease pathogenesis. In patients with inflammatory myopathies, although RTX‐mediated B‐cell depletion has shown significant efficacy, some cases remain refractory, possibly because of incomplete depletion of plasma cells and tissue‐resident B cells. Therefore, CAR‐T‐cell therapy has been used as a potential treatment option for IIM.

In IIM, CD19 CAR‐T‐cell therapy has been successfully applied in several patients with refractory ASS, resulting in marked improvement in both muscle and extramuscular manifestations, with sustained drug‐free remission during follow‐up, with the longest follow‐up period reaching 18 months [25, 26, 27]. Another study proposed combining CAR‐T‐cell therapy with T‐cell suppression (Mycophenolate mofetil，MMF) to induce sustained drug‐free remission in patients with ASS [28]. Both CD19 and BCMA CAR‐T‐cell therapies have shown promising efficacy in treating refractory IMNM [29, 30]. Recently, the first case of JDM treated with CD19 CAR‐T‐cell therapy was reported, showing gradual improvement in both muscle and skin involvement. Sustained drug‐free remission in IIM following CAR‐T‐cell therapy is hypothesized to result from complete B‐cell depletion and subsequent reconstitution of a B‐cell system lacking pathogenic B cells [31]. Given the high cost and complex manufacturing process of autologous CAR‐T‐cell therapy, allogeneic CAR‐T‐cell therapy has been explored in patients with IMNM, resulting in significant clinical improvement and B‐cell depletion without graft‐versus‐host disease [30]. However, a recent case report described a patient with refractory AAS who achieved disease remission following treatment with CD19‐directed CAR‐T‐cell therapy; however, disease relapse occurred 9 months later. Reinfusion of the same CAR‐T‐cell mixture failed to induce expansion. This case highlights the ongoing challenges associated with CAR‐T‐cell reinfusion [32].

Additionally, CAR‐T‐cell therapy has demonstrated favourable safety and tolerability profiles in IIM patients. Common adverse events include leukopenia, mild cytokine release syndrome (CRS), immune effector cell‐associated neurotoxicity syndrome (ICANS) and infections, with no life‐threatening adverse reactions observed to date. CRS and ICANS require management according to clinical guidelines. Regular monitoring of immunoglobulin levels with replacement therapy when indicated is essential during treatment. Furthermore, the long‐term immunological consequences and characteristics of potential late‐onset toxicities remain incompletely understood. To mitigate these risks, a comprehensive pretherapeutic assessment of organ function and infectious status, along with vigilant long‐term monitoring for signs of infection and haematological abnormalities, is imperative [33].

## 4 BiTE

The T‐cell engager teclistamab acts on T cells via CD3, and targets plasmablasts and plasma cells through BCMA. Teclistamab has demonstrated considerable efficacy in patients with multiple myeloma. In a case series, teclistamab was administered to four patients with autoimmune diseases who were resistant to multiple immunosuppressive agents, including RTX. This cohort included one patient with MDA5‐positive DM. Following treatment, the MDA5‐positive DM patient exhibited significant reduction in cutaneous inflammation and ulceration. During therapy, a transient peak in CRP and inflammatory cytokines was observed between Days 2 and 5, without the occurrence of severe adverse events. These findings suggest that BCMA‐targeted T‐cell engagers, represent a feasible therapeutic approach for patients with autoimmune disorders. Further exploration of such agents in the treatment of IIM may be warranted in the future [34].

## 5 T‐Cell–Targeting Therapies

Abatacept, a recombinant fusion protein of CTLA‐4, inhibits T‐cell activation by blocking the interaction between CD80/CD86 and CD28. It has been approved by the FDA for the treatment of rheumatoid arthritis in both adults and children [35]. Studies have shown that abatacept can increase the frequency of regulatory FOXP3+ T cells and promote the production of amphiregulin, which may facilitate myofiber regeneration [36]. In a murine C‐protein‐induced myositis (CIM) model, treatment with CTLA4‐Ig or anti‐CD80/86 antibodies effectively suppressed myositis, suggesting its potential therapeutic feasibility for myositis [37].

Clinical studies further support the application of abatacept in IIM. A study involving 20 patients with refractory DM (*n* = 9) or PM (*n* = 11) demonstrated that approximately half of the patients experienced a reduction in disease activity after 6 months of abatacept treatment [38]. Recently, a Phase Ⅲ randomized controlled trial evaluated abatacept in combination with standard therapy for active IIM. The results revealed that the abatacept and placebo groups had comparable safety profiles and similar proportions of patients meeting the International Myositis Assessment and Clinical Studies (IMACS) improvement criteria at 24 weeks. Subgroup analysis revealed a greater proportion of patients who achieved improvement criteria in the non‐DM subgroup treated with abatacept, suggesting that abatacept may provide more sustained benefits for non‐DM subtypes [39]. Additionally, a retrospective study involving eight patients with ASS‐associated ILD revealed that abatacept alleviated symptoms in all patients without any observed treatment‐related adverse events [40]. Abatacept has also been explored in JDM. A clinical study of 10 JDM patients with moderate disease activity revealed that 9 patients met the disease improvement criteria at week 24. All core set measures (CSMs), except for muscle enzymes, improved from baseline, and a significant glucocorticoid‐sparing effect was observed. However, 11 Grade 2 or 3 treatment‐related adverse events were reported during the study [35]. Notably, calcinosis, which is common in JDM and significantly impacts patients' quality of life, may also be ameliorated with abatacept treatment [41].

In conclusion, current studies indicate the potential efficacy and safety of abatacept in the treatment of IIM. Further research is needed to explore its application in specific disease subtypes and optimize treatment strategies, aiming to improve disease remission rates while minimizing adverse events.

## 6 B‐ and T‐Cell–Targeting Therapies

Alemtuzumab, a humanized monoclonal antibody against CD52, depletes B and T cells by binding to CD52 on lymphocytes. In a refractory case of anti‐Jo‐1‐positive PM with ILD, a single cycle of the drug improved muscle symptoms and enabled glucocorticoid tapering but did not prevent respiratory decline, with the patient dying 1 year post‐treatment. This finding indicates that its role in IIM has not yet been proven and that therapy must be personalized according to organ involvement [42].

## 7 TNF Inhibitors

Tumour necrosis factor‐*α* (TNF‐*α*) is a pleiotropic cytokine secreted by immune cells that is characterized by its potent proinflammatory activity and is involved in the pathogenesis of IIM. However, the application of TNF‐*α* inhibitors in the treatment of IIM remains controversial.

In a case series, five patients with refractory DM were reported to experience disease exacerbation following the administration of etanercept [43]. In contrast, two randomized, double‐blind, placebo‐controlled trials demonstrated that etanercept facilitated glucocorticoid tapering, significantly slowed disease progression, and was well tolerated [44]. Another randomized, double‐blind, placebo‐controlled trial enrolled 12 patients with DM or PM presenting with muscle weakness. Compared with the placebo, the addition of infliximab to stable doses of immunosuppressants and glucocorticoids resulted in improved muscle strength [46]. Furthermore, a cohort study involving 60 JDM patients revealed that treatment with infliximab or adalimumab for at least 3 months improved both muscle and skin manifestations. Among these patients, those who switched from infliximab to adalimumab experienced further reductions in overall disease activity [47]. Infliximab has also been shown to be effective against calcinosis in refractory JDM patients [48, 49]. Case reports suggest that adalimumab may be beneficial for DM patients with ILD who respond poorly to conventional therapies [100, 101]. Similarly, early administration of infliximab in DM patients with acute interstitial pneumonia demonstrated favourable outcomes, indicating the potential therapeutic value of TNF‐*α* inhibitors in the treatment of IIM‐associated ILD.

However, findings regarding the efficacy of TNF‐*α* inhibitors are inconsistent. In a study involving 13 patients with refractory IIM, nine patients completed four infusions of infliximab, while the others discontinued treatment due to adverse events or the detection of malignancies. Follow‐up of the treated patients revealed persistent infiltration of T lymphocytes and macrophages, cytokine expression and increased Type I interferon activity in muscle biopsies after treatment, indicating a lack of therapeutic efficacy [50]. Another study revealed no significant improvement in patients with refractory JDM treated with etanercept, with some patients experiencing worsening of disease manifestations, such as aggravated rashes [102]. There have also been reports of TNF‐*α* inhibitors inducing inflammatory myopathies in patients with other autoimmune diseases, such as rheumatoid arthritis [45].

The use of TNF‐*α* inhibitors in IIM is complicated by a paradoxical risk of inducing or exacerbating autoimmune phenomena, including the onset of myositis. Additionally, they confer an elevated risk of serious infections, particularly opportunistic infections and reactivation of latent tuberculosis, necessitating rigorous screening and antimicrobial prophylaxis. The potential for increased malignancy risk, although not conclusively established in IIM populations, warrants caution. The benefit–risk balance must be carefully evaluated, with particular consideration given to the patient's specific clinical phenotype, prior history of malignancy, and comprehensive infectious disease screening. In summary, the efficacy of TNF‐*α* inhibitors in IIM requires further validation through large‐scale randomized controlled trials.

## 8 IFN‐I Receptor Antagonist

Type I IFN (IFN‐I) is a critical pathway in the pathogenesis of IIM, contributing to disease progression by inducing endothelial cell and muscle fibre damage. Studies have demonstrated that IFN‐I‐induced sarcoplasmic MxA expression is significantly associated with the muscle disease activity of JDM patients [103]. Furthermore, the IFN‐I pathway has been implicated in the upregulation of MHC‐I in DM, which sustains autoimmune inflammatory responses. Additionally, IFN‐I exposure has been shown to cause muscle atrophy, accompanied by the upregulation of atrophy‐related genes, such as Atrogin‐1 and Murf‐1, suggesting that muscle atrophy in DM patients may be linked to the activation of the IFN‐I pathway.

Anifrolumab, a type I IFN antagonist, blocks IFN‐I activity by binding to subunit 1 of the type I IFN receptor. It has been approved for the treatment of systemic lupus erythematosus (SLE) and has shown preliminary evidence of efficacy in the treatment of IIM. In a case report, a 14‐year‐old girl with refractory disease who failed 6 years of treatment with tofacitinib and glucocorticoids showed significant improvement in skin rash within 72 h of the first infusion of anifrolumab, indicating the potential for rapid disease control [51]. Another multicenter retrospective study included seven patients with refractory DM, all of whom presented with predominant skin involvement, and three had a history of severe muscle damage. Significant improvement in skin disease activity was observed after anifrolumab treatment, with a mean reduction in the Cutaneous Disease Area and Severity Index (CDASI) score of 13.0 after two doses. The main adverse events were infections, including upper respiratory tract infections in two patients and peritonsillar abscess in one patient [52]. Concurrently, a case series involving four patients with refractory DM demonstrated the potential of anifrolumab in addressing refractory extramuscular manifestations, facilitating prednisone tapering, and enabling discontinuation of systemic therapy [53].

Therefore, anifrolumab shows promise in the treatment of refractory cutaneous manifestations of DM, although its efficacy and safety require further validation.

## 9 IL‐6 Receptor Antagonist

Interleukin‐6 (IL‐6) is closely associated with muscle inflammation and disease activity in patients with IIM. Tocilizumab is a monoclonal antibody that targets the IL‐6 receptor and was first used in 2011 in two patients with refractory ASS. Following treatment, both patients demonstrated significant improvements in joint, muscle, and pulmonary involvement. After more than 10 years of treatment, both patients remained in complete remission, indicating that IL‐6 inhibition might represent a long‐term therapeutic option for refractory ASS [54]. A patient with NXP2‐positive DM experienced recurrence of rash and edema after cyclophosphamide and methylprednisolone pulse therapy. After three cycles of tocilizumab, the patient showed marked improvement in rashes and recovery of muscle strength. After six cycles of treatment, there was no recurrence of rash or edema, and the dosage of glucocorticoids was gradually tapered [55]. An open‐label study involving 11 patients with refractory IMNM revealed that 63.6% of patients achieved significant clinical improvement on the basis of the 2016 ACR‐EULAR myositis response criteria following tocilizumab treatment. Baseline serum IL‐6 levels and the percentage of CD56‐positive muscle fibres were positively correlated with the total improvement score (TIS) after 6 months of treatment, potentially serving as predictive factors for the response to tocilizumab [56]. Tocilizumab has also been reported to be effective in patients with MDA5‐positive DM or JDM complicated by rapid‐progressive ILD (RP‐ILD) [57, 58]. In a clinical study, six patients with anti‐MDA5‐positive DM complicating RP‐ILD who were refractory to conventional therapy received tocilizumab treatment. Significant improvements in respiratory symptoms and imaging findings were observed in five patients, indicating that tocilizumab may be a salvage therapy for patients with RP‐ILD refractory to intensive immunosuppressive regimens [59].

However, in a multicenter, randomized, double‐blind, placebo‐controlled trial involving 36 patients with refractory adult DM and PM, no significant difference in the TIS was observed between the tocilizumab and placebo groups at 24 weeks. Secondary endpoints, including time to improvement, time to worsening, changes in core set measures, safety outcomes, and glucocorticoid‐sparing effects, also showed no significant differences between the two groups. Since the randomized trial of tocilizumab in the treatment of refractory DM/PM failed to meet its primary endpoint, tocilizumab cannot be recommended as a routine treatment option. However, it may serve as an effective salvage therapy for patients with refractory ASS and MDA5‐positive DM complicated by RP‐ILD [60].

## 10 IL‐1 Receptor Antagonists

Elevated expression of IL‐1*α* and IL‐1*β* in muscle tissue has been confirmed in patients with DM and PM. IL‐1 contributes to disease pathogenesis by inducing the expression of inflammation‐related genes through receptor‐mediated signalling [104]. Anakinra, an IL‐1 receptor antagonist, reduces the levels of IL‐1*β* in muscle fibres, thereby decreasing its colocalization with amyloid precursor protein. This subsequently reduces the deposition of *β*‐amyloid and misfolded proteins, leading to disease amelioration [61]. In a preclinical study, a CIM model revealed significantly increased mRNA and protein levels of IL‐1*α*, IL‐1*β* and TNF‐*α* in muscle tissue. Antagonism of the IL‐1 receptor effectively suppressed the development of myositis, suggesting the potential therapeutic application of IL‐1 receptor blockade in myositis treatment [62].

The clinical efficacy of anakinra has been further validated. An open‐label study involving 15 patients with refractory myositis demonstrated that seven patients achieved clinical responses after 12 months of anakinra treatment, with no significant adverse events observed. These findings highlight the favourable safety profile of anakinra [63]. The study also revealed that patients with higher extramuscular involvement scores were more likely to respond to anakinra. Additionally, case reports have shown that anakinra provides rapid and significant improvements in general and extramuscular manifestations in patients with refractory MDA5‐positive DM [64]. In another study, anakinra was administered to five patients with ASS, resulting in significant reductions in C‐reactive protein and troponin T levels. All patients achieved the major improvement criteria defined by the IMACS group, with no adverse events observed during follow‐up [65].

However, studies on IBM have shown limited efficacy. Four patients with biopsy‐confirmed IBM who received anakinra for an average of 7.7 months exhibited no improvement in muscle strength or stability [61]. Similarly, canakinumab, an IL‐1*β* receptor antagonist, has demonstrated no significant efficacy in IBM patients [66].

In conclusion, anakinra may be effective for myositis, particularly in patients with prominent extramuscular involvement, but it is ineffective for the treatment of IBM. Further studies are needed to optimize its dosage and administration and evaluate its therapeutic efficacy.

## 11 Fc Receptor‐Targeting Therapies

Efgartigimod is a human IgG1 antibody fragment that binds to the neonatal Fc receptor (FcRn) and competitively inhibits the interaction between IgG and FcRn, thereby promoting the lysosomal degradation of IgG and reducing its recycling [67]. Currently, it is primarily used for the treatment of myasthenia gravis, where it significantly lowers the serum IgG levels of pathogenic acetylcholine receptor antibodies [68]. In a humanized mouse model of IMNM, efgartigimod effectively reduced circulating IgG levels, including pathogenic anti‐HMGCR IgG antibodies, prevented muscle fibre necrosis and promoted muscle fibre regeneration [67]. A subsequent clinical investigation enrolled seven patients with refractory IMNM who received efgartigimod treatment for 4 weeks. The results demonstrated a marked reduction in the serum IgG and anti‐HMGCR/SRP antibody levels compared with those at baseline, with four patients achieving clinical remission. Throughout the treatment period, only one adverse event, mild headache, was reported [69]. These findings indicate that efgartigimod has promising therapeutic potential and demonstrates favourable safety in the treatment of IIM.

## 12 JAK Inhibitors

JAK inhibitors are a class of small‐molecule targeted drugs designed to block the JAK/STAT signalling pathway by competitively binding to ATP, thereby suppressing proinflammatory cytokine activation, alleviating inflammation, and treating diseases [33]. The JAK family includes JAK1, JAK2, JAK3 and TYK2, with major drugs including tofacitinib, baricitinib, upadacitinib and ruxolitinib.

### 12.1 Tofacitinib

Tofacitinib reduces the levels of anti‐Jo1 autoantibodies, CXCL10, and CXCL13 in memory B‐cell cultures derived from patients with ASS. These findings position the JAK/STAT pathway as a promising novel therapeutic target for modulating B‐cell activity in IIM [70]. A retrospective study demonstrated that tofacitinib significantly improved skin symptoms in patients with classic DM and amyopathic DM (ADM) but had limited effects on muscle strength in patients with DM or PM; individual patients may experience adverse reactions such as viral infections, EBV‐positive central nervous system lymphoma, and severe headache [71]. A prospective open‐label study involving 10 patients with refractory active DM revealed that all participants met the disease improvement criteria defined by the IMACS at week 12 [72]. A Chinese study revealed that, compared with conventional therapy, tofacitinib combined with glucocorticoids improved the 6‐month survival rate in MDA5‐positive DM patients with ILD. Additionally, serum ferritin levels, forced vital capacity (FVC), single‐breath diffusing capacity for carbon monoxide and high‐resolution CT findings were significantly improved, with most adverse events being mild [73]. Another study revealed that increasing the dose of tofacitinib from 10 mg/d to 20 mg/d could improve symptoms in patients with MDA5‐positive DM who were unresponsive to 10 mg/d tofacitinib [71]. However, this approach requires careful evaluation of the risk–benefit balance due to the increased risk of infections. In JDM, interferons and Janus kinases play critical roles in pathogenesis. A retrospective analysis revealed that tofacitinib improved skin manifestations, muscle symptoms, ILD, calcinosis and medication tapering in JDM patients [74, 75].

### 12.2 Baricitinib

Type I IFN contributes to DM pathogenesis by upregulating the expression of interferon‐stimulated genes (ISGs) in the blood and activating p‐JAK1 and p‐STAT1 in the skin. Baricitinib, a JAK1/JAK2 inhibitor, exerts therapeutic effects on IIM by suppressing Type I IFN‐induced myocyte and microvascular damage. A case report revealed that baricitinib improved the condition of three DM patients who were unresponsive to conventional therapies. In vitro studies revealed significant downregulation of IFI44 and IFI27 expression, confirming that baricitinib reduces ISG levels [76]. Another study involving 12 DM patients demonstrated that baricitinib significantly improved facial erythema and reduced the levels of CXCL10, CCL2 and galectin‐9. Baricitinib combined with traditional immunosuppressants was effective in treating relapsing JDM, with improvements in skin and muscle symptoms and no severe adverse events observed [77]. A single‐centre retrospective study demonstrated that baricitinib significantly improved cutaneous manifestations in children with refractory JDM who had inadequate responses to corticosteroids and immunosuppressants. Beneficial effects were also observed in calcinosis and ILD, with concurrent resolution of macrophage activation syndrome. Treatment was well tolerated, with only one patient discontinued due to herpes zoster infection; no other significant adverse events or mortality were reported [77, 78]. A randomized controlled clinical trial demonstrated that baricitinib exhibits superior efficacy over RTX in the treatment of refractory MDA5‐positive DM, which is attributable to its capacity to concurrently suppress the interferon and GM‐CSF signalling pathways. This dual inhibition effectively modulates macrophage‐mediated hyperactive autoimmune responses, highlighting its therapeutic advantage in this challenging patient population [79, 80]. Currently, randomized controlled trials are underway to evaluate baricitinib in refractory and relapsing DM patients.

### 12.3 Upadacitinib

Retrospective studies have shown that upadacitinib significantly improves skin symptoms in refractory DM and ASS patients, but its effects on muscle strength could not be assessed because of the lack of active muscle disease at baseline [81]. Another study demonstrated that upadacitinib effectively alleviated skin lesions in refractory DM patients and may help prevent disease recurrence [82].

### 12.4 Ruxolitinib

Ruxolitinib, a JAK1/JAK2 inhibitor, has shown potential in the treatment of DM. A case report described a female patient with postpolycythemia vera myelofibrosis and DM who experienced significant improvement in DM symptoms during ruxolitinib treatment, suggesting its potential as a therapeutic option for DM [83]. Studies have also shown that ruxolitinib reduces IFN activity in JDM patients and improves skin lesions, muscle weakness and serum type I IFN levels in refractory DM patients [84, 85]. A meta‐analysis revealed that JAK inhibitors significantly reduced CDASI in IIM patients, with ruxolitinib resulting in the most pronounced CDASI reduction [86].

In conclusion, JAK inhibitors show potential for improving outcomes in IIM patients who are refractory to conventional therapies. However, JAK inhibitors are associated with an increased risk of serious infections, including herpes zoster reactivation and opportunistic infections, which may warrant prophylactic strategies. Cardiovascular and thromboembolic risks, particularly in patients with underlying risk factors, have been highlighted in other inflammatory conditions. Additionally, laboratory monitoring for cytopenias and lipid profile alterations is recommended. The benefit–risk assessment must account for the patient's age, cardiovascular and thrombotic risk profile and vaccination status against herpes zoster.

## 13 Proteasome Inhibitors

Zetomipzomib (KZR‐616) is a selective immunoproteasome inhibitor that targets the LMP7 and LMP2 subunits of the immunoproteasome. It reduces the production of proinflammatory cytokines, inhibits plasma cell activity and decreases the production of autoantibodies, thereby showing broad therapeutic potential in various autoimmune diseases [87]. An animal experiment revealed that KZR‐616 treatment prevented the loss of grip strength in CIM mice, reduced leukocyte infiltration in muscle tissue, and inhibited the elevation of serum creatine kinase levels. On the basis of these preclinical findings, researchers further conducted clinical trials of KZR‐616. In a randomized, double‐blind, placebo‐controlled trial involving 25 IIM patients, KZR‐616 exhibited favourable safety profiles. However, no significant differences in efficacy were observed between the treatment and placebo groups [88]. Further studies are warranted to elucidate the therapeutic potential of KZR‐616 in IIM.

## 14 Low‐Dose IL‐2 Therapy

Low‐dose IL‐2 therapy exerts its therapeutic effects by promoting the expansion and activation of regulatory T cells (Tregs) while suppressing effector T‐cell (Teff) activity, thereby attenuating muscle tissue inflammation. Additionally, low‐dose IL‐2 reduces chemokine secretion by fibroblasts and inhibits peripheral lymphocyte migration, further mitigating muscle damage and inflammatory cascade formation [89].

A clinical study involving 71 patients with DM/PM and 30 HCs demonstrated that compared with HCs, PM/DM patients presented significantly lower absolute numbers and proportions of peripheral blood Tregs, elevated Th17/Treg ratios and reduced IL‐2 production. Low‐dose IL‐2 treatment significantly increased Treg numbers [90]. In an expanded cohort of 147 PM/DM patients, comprising 116 patients receiving conventional therapy and 31 receiving conventional therapy plus low‐dose IL‐2, combination therapy resulted in an approximately fourfold increase in absolute Treg numbers and significant improvement in disease parameters, validating the efficacy of low‐dose IL‐2 treatment [89].

In a prospective cohort study, 18 patients with active IIM received low‐dose IL‐2 treatment for 12 weeks. Among them, 77.78% (14/18) achieved disease remission according to the IMACS criteria, and 83.33% (15/18) met the 2016 ACR/EULAR myositis response criteria. Additionally, patients demonstrated significant improvement in cutaneous manifestations. No significant adverse events were observed during the treatment period, indicating both the efficacy and safety of low‐dose IL‐2 therapy in patients with IIM [91]. Additionally, a case report confirmed the efficacy of low‐dose IL‐2 in treating hypomyopathic DM with refractory dermatitis [92]. Another study revealed that low‐dose IL‐2 therapy helps to restore the Th17/Treg balance in DM patients complicated by EBV/CMV viremia [93]. To further explore the mechanism underlying the use of IL‐2 in the treatment of IIM, researchers discovered that it can exert a therapeutic effect by modulating the dysregulated gut microbiome of IIM patients [94]. Therefore, the combination of low‐dose IL‐2 with conventional therapy significantly increases Treg cell numbers and maintains immune homeostasis, potentially alleviating symptoms in DM/PM patients while enhancing their anti‐infection capabilities.

## 15 Discussion and Conclusion

IIM represents a highly heterogeneous group of autoimmune diseases with unclear aetiology and complex, diverse clinical and histopathological manifestations. Even with first‐line treatments such as glucocorticoids, a subset of patients exhibits a refractory and relapsing course. The development of targeted therapies has transformed the treatment landscape for IIM, offering hope to patients with refractory disease or severe complications. This review synthesizes the current evidence on targeted therapies for IIM, affirming their potential to induce remission in refractory patients while highlighting significant challenges, including variability in efficacy and safety concerns.

B‐cell‐targeted therapies represent a relatively mature treatment paradigm. RTX has demonstrated significant efficacy in reducing disease activity, particularly in patients with ASS and ILD. Compared with conventional therapies, RTX has comparable or superior efficacy and a favourable safety profile, positioning it as one of the preferred treatments for IIM. Supported by meta‐analysis evidence and formal guidelines, RTX has become a cornerstone therapy for refractory disease. Furthermore, the timing of RTX administration is critical, with early intervention associated with improved prognosis. The emergence of next‐generation anti‐CD20 agents, such as obinutuzumab, provides novel options for patients who are resistant or intolerant to RTX. Additionally, drugs that target plasma cells, such as daratumumab, represent a new direction towards deeper depletion of the B‐cell lineage. In contrast, the value of belimumab‐induced BAFF inhibition in IIM remains uncertain because primary endpoints are not met in RCTs, suggesting that not all B‐cell pathways hold equivalent therapeutic relevance in this disease.

T‐cell‐targeted therapies exhibit subtype‐dependent efficacy. Abatacept has unique value in nondermatomyositis subtypes and JDM‐associated calcinosis, although its benefits in classic DM remain limited.

Targeted therapies against specific cytokine pathways have achieved notable successes but are accompanied by thought‐provoking contradictions. The JAK–STAT pathway has been identified as a critical therapeutic target. JAK inhibitors, particularly tofacitinib and baricitinib, show remarkable efficacy in ameliorating refractory cutaneous manifestations. Notably, baricitinib even showed superior efficacy compared with RTX in an RCT for MDA5‐positive DM‐associated ILD, which was attributed to its dual inhibition of the interferon and GM‐CSF signalling pathways. Similarly, the rapid and significant improvement of cutaneous symptoms with anifrolumab in refractory DM and JDM provides strong evidence that the type I interferon pathway is a key driver of specific clinical phenotypes. Although tocilizumab failed to meet primary endpoints in an RCT for refractory DM/PM, it remains an important salvage therapy for refractory ASS‐ and MDA5‐positive DM patients with rapidly progressive ILD. This variability underscores the importance of patient stratification on the basis of clinical phenotypes and potential biomarkers, such as serum IL‐6 levels and CD56‐positive muscle fibres. The IL‐1 receptor antagonist anakinra shows efficacy in IIM patients, particularly those with prominent extra‐articular organ involvement, but clinical studies indicate no significant benefit in IBM, further reinforcing the concept of phenotype‐driven therapy. The use of TNF‐*α* inhibitors in IIM remains highly controversial, with reports of improvement in cutaneous and muscular symptoms as well as calcinosis contrasted with studies showing inefficacy or even disease exacerbation. These findings reveal the complex and dual role of cytokines in IIM. The heterogeneity of treatment responses across and within subtypes poses a central challenge, largely because of the diversity of immunopathogenic mechanisms.

Novel mechanisms are expanding therapeutic options. Efgartigimod, by targeting the Fc receptor, offers a new strategy to reduce pathogenic IgG autoantibodies and shows promising results in IMNM. Low‐dose IL‐2 therapy, which aims to restore immune balance by selectively expanding regulatory T cells, has been shown to improve disease activity and is attractive for its potential to enhance anti‐infective capacity. Similarly, bispecific T‐cell engagers (BiTEs), such as teclistamab, which targets BCMA and CD3, represent a novel therapeutic strategy by recruiting and activating a patient's own T cells to eliminate plasma cells and plasmablasts precisely. However, this potent mode of T‐cell activation is associated with inherent risks, including CRS, necessitating further clinical studies to fully elucidate its efficacy and safety profile.

In recent years, CAR‐T‐cell therapy has emerged as a research hotspot in autoimmune disease treatment. By enabling deep B‐cell depletion and inducing profound immune reset, CAR‐T‐cell therapy has shown remarkable efficacy, achieving sustained drug‐free remission in patients with refractory ASS, IMNM and JDM. However, reports of relapse and reinfusion failure, alongside unique adverse events such as CRS, indicate significant challenges in long‐term efficacy and toxicity management.

Despite the exciting progress in targeted therapies for IIM, several systemic challenges remain. First, the existing evidence exhibits significant heterogeneity. Many conclusions are derived from small open‐label studies or case series, which carry inherent risks and have limited generalizability. Inconsistent evidence for therapies such as TNF inhibitors and belimumab necessitates caution and highlights the urgent need for large‐scale, high‐quality RCTs. Second, while adverse events for each therapy have been discussed, deeper risk–benefit stratification is needed. For example, the clinical application of CAR‐T cells and JAK inhibitors demands infection prophylaxis and long‐term monitoring. Third, the high cost and implementation complexity of advanced therapies such as CAR‐T cells currently limit their widespread use, despite their revolutionary potential to induce sustained, drug‐free remission.

Finally, the inability to perform quantitative synthesis (meta‐analysis) in this review reflects the current literature landscape: significant clinical and methodological heterogeneity among numerous small studies makes data pooling difficult. Future accumulation of more homogeneous data may enable systematic reviews and meta‐analyses to provide higher‐level evidence.

In conclusion, targeted therapies are revolutionizing the management of IIM. B‐cell‐targeted agents, T‐cell modulators, cytokine inhibitors, CAR‐T‐cell therapies and proteasome inhibitors have demonstrated efficacy in reducing disease activity and improving clinical outcomes. However, challenges remain in optimizing treatment, managing adverse events, developing novel targeted drugs and conducting larger clinical trials. By addressing these challenges, targeted therapies have the potential to significantly enhance the quality of life and long‐term prognosis of patients with IIM.

## Funding

This study was supported by Sichuan Science and Technology Program (2025ZNSFSC0638 [G.Y.] and 2024YFFK0062 [Q.B.X.]). This study was also supported by the National Natural Science Foundation of China (No. 82572070).

## Ethics Statement

The manuscript does not contain original clinical studies or patient data. All the authors listed in the manuscript comply with the ethical guidelines for authorship and publishing in the *Journal of Cachexia, Sarcopenia and Muscle*.

## Conflicts of Interest

The authors declare no conflicts of interest.

## References

[1] T. Khoo , J. B. Lilleker , B. Y. H. Thong , V. Leclair , J. A. Lamb , and H. Chinoy , “Epidemiology of the Idiopathic Inflammatory Myopathies,” Nature Reviews Rheumatology 19, no. 11 (2023): 695–712. 37803078 10.1038/s41584-023-01033-0

[2] I. E. Lundberg , M. Fujimoto , J. Vencovsky , et al., “Idiopathic Inflammatory Myopathies,” Nature Reviews. Disease Primers 7, no. 1 (2021): 87. 10.1038/s41572-021-00325-7 PMC10425161 34857780

[3] K. Nagaraju , S. Ghimbovschi , S. Rayavarapu , et al., “Muscle Myeloid Type I Interferon Gene Expression May Predict Therapeutic Responses to Rituximab in Myositis Patients,” Rheumatology (Oxford) 55, no. 9 (2016): 1673–1680. 27215813 10.1093/rheumatology/kew213 PMC5854096

[4] T. M. Maher , V. A. Tudor , P. Saunders , et al., “Rituximab Versus Intravenous Cyclophosphamide in Patients With Connective Tissue Disease‐Associated Interstitial Lung Disease in the UK (RECITAL): A Double‐Blind, Double‐Dummy, Randomised, Controlled, Phase 2b Trial,” Lancet Respiratory Medicine 11, no. 1 (2023): 45–54. 36375479 10.1016/S2213-2600(22)00359-9

[5] R. Aggarwal , “Rituximab in Myositis,” Rheumatology (Oxford) 50, no. 12 (2011): 2155–2156. 21571769 10.1093/rheumatology/ker138

[6] C. Campochiaro , N. Farina , G. de Luca , et al., “Effectiveness and Safety of Mycophenolate Mofetil and Rituximab Combination Therapy for Immune Idiopathic Myopathies,” Arthritis Research & Therapy 26, no. 1 (2024): 79. 38570792 10.1186/s13075-024-03310-z PMC10988925

[7] V. Leclair , A. S. Galindo‐Feria , M. Dastmalchi , M. Holmqvist , and I. E. Lundberg , “Efficacy and Safety of Rituximab in Anti‐Synthetase Antibody Positive and Negative Subjects With Idiopathic Inflammatory Myopathy: A Registry‐Based Study,” Rheumatology (Oxford) 58, no. 7 (2019): 1214–1220. 30690633 10.1093/rheumatology/key450

[8] J. Narváez , E. Cañadillas , I. Castellví , et al., “Rituximab in the Treatment of Progressive Interstitial Lung Disease Associated With the Antisynthetase Syndrome,” Arthritis Research & Therapy 26, no. 1 (2024): 122. 38890654 10.1186/s13075-024-03353-2 PMC11184916

[9] M. Peskin , M. Mostowy , J. Velez , M. Perron , J. Kurian , and D. M. Wahezi , “Clinical Improvement in Early Onset Interstitial Lung Disease Using Rituximab in Children With Antimelanoma Differentiation‐Associated Gene 5‐Positive Juvenile Dermatomyositis,” Journal of Rheumatology 51 (2023): 69–74. 10.3899/jrheum.2023-0544 37714547

[10] A. Manwatkar , K. Naresh , J. Mathew , et al., “Comparison of Rituximab Efficacy in Treatment‐Naive and Refractory Inflammatory Myopathies: Experiences From a Tertiary Care Centre,” Rheumatology (Oxford) 64 (2024): 2091–2098. 10.1093/rheumatology/keae307 38814804

[11] P. Mehta , R. Dorsey‐Campbell , P. Dassan , C. Nelson‐Piercy , and S. Viegas , “Difficult Case: Rituximab in Anti‐SRP Antibody Myositis in Pregnancy,” Practical Neurology 19, no. 5 (2019): 444–446. 30979789 10.1136/practneurol-2018-002168

[12] R. Janardana , S. N. Amin , L. Rajasekhar , et al., “Low‐Dose Rituximab Is Efficacious in Refractory Idiopathic Inflammatory Myopathies,” Rheumatology (Oxford) 62, no. 3 (2023): 1243–1247. 35946502 10.1093/rheumatology/keac438

[13] C. Zhen , Y. Hou , B. Zhao , X. Ma , T. Dai , and C. Yan , “Efficacy and Safety of Rituximab Treatment in Patients With Idiopathic Inflammatory Myopathies: A Systematic Review and Meta‐Analysis,” Frontiers in Immunology 13 (2022): 1051609. 36578492 10.3389/fimmu.2022.1051609 PMC9791086

[14] M. Barré , E. Delaporte , P. Berbis , and M. Benzaquen , “Severe Dermatomyositis Revealing a Thymic Carcinoma: Did Rituximab Delay the Diagnosis?,” Dermatologic Therapy 33, no. 6 (2020): e14016. 32667098 10.1111/dth.14016

[15] A. Alqahtani , M. Sabha , T. Abdelfattah , et al., “Tendonitis and Tendon Rupture After Treatment With Rituximab: A Case Series,” American Journal of Therapeutics 24, no. 5 (2017): e592–e595. 28418945 10.1097/MJT.0000000000000591

[16] M. Tokunaga , Y. Nakai , Y. Sato , et al., “A Pilot Transcriptomic Study of a Novel Multitargeted BRT Regimen for Anti‐MDA5 Antibody‐Positive Dermatomyositis: Improving Survival over Conventional Therapy,” Frontiers in Immunology 16 (2025), 1568338, 10.3389/fimmu.2025.1568338. 40852724 PMC12367743

[17] M. Groener and J. Paik , “Emerging B and Plasma Cell‐Targeting Immune Therapies in Idiopathic Inflammatory Myopathies,” Frontiers in Immunology 16 (2025): 1581323. 40746557 10.3389/fimmu.2025.1581323 PMC12310725

[18] A. N. Stütz , P. Kvacskay , C. P. Heußel , et al., “Efficacy of Obinutuzumab in two Rituximab Refractory Cases of Idiopathic Inflammatory Myopathies With Severe Interstitial Lung Disease,” Annals of the Rheumatic Diseases (2025): S0003‐4967(25)04236‐0. 10.1016/j.ard.2025.07.004 40744769

[19] I. Ilic and G. Marder , “Experience on the Use of Obinutuzumab off Label in Patients with Refractory Idiopathic Inflammatory Myopathy,” Arthritis & Rheumatology 76 (2024): 4197–4198.

[20] P. Kvacskay , W. Merkt , J. G. Günther , et al., “Obinutuzumab in Connective Tissue Diseases After Former Rituximab‐Non‐Response: A Case Series,” Annals of the Rheumatic Diseases 81, 5 (2022): 744–746. 35027400 10.1136/annrheumdis-2021-221756 PMC8995802

[21] A. Baek , H. J. Park , S. J. Na , et al., “The Expression of BAFF in the Muscles of Patients With Dermatomyositis,” Journal of Neuroimmunology 249, no. 1–2 (2012): 96–100. 22575417 10.1016/j.jneuroim.2012.04.006

[22] P. O. Carstens , L. M. Müllar , A. Wrede , et al., “Skeletal Muscle Fibers Produce B‐Cell Stimulatory Factors in Chronic Myositis,” Frontiers in Immunology 14 (2023): 1177721. 37731487 10.3389/fimmu.2023.1177721 PMC10508232

[23] Y. Liu , Y. Li , T. Shen , et al., “Belimumab Ameliorates Symptoms and Disease Activity in Patients With Dermatomyositis and Juvenile Dermatomyositis Refractory to Standard Therapy: A Retrospective Observational Study,” Journal of the American Academy of Dermatology 91, no. 3 (2024): 524–527. 38697217 10.1016/j.jaad.2024.04.060

[24] G. Marder , T. Quach , P. Chadha , et al., “Belimumab Treatment of Adult Idiopathic Inflammatory Myopathy,” Rheumatology (Oxford) 63, no. 3 (2024): 742–750. 37326854 10.1093/rheumatology/kead281 PMC10907809

[25] J. Taubmann , J. Knitza , F. Müller , et al., “Rescue Therapy of Antisynthetase Syndrome With CD19‐Targeted CAR‐T Cells After Failure of Several B‐Cell Depleting Antibodies,” Rheumatology (Oxford) 63, no. 1 (2024): e12–e14. 37432378 10.1093/rheumatology/kead330 PMC10765150

[26] F. Müller , S. Boeltz , J. Knitza , et al., “CD19‐Targeted CAR T Cells in Refractory Antisynthetase Syndrome,” Lancet 401, no. 10379 (2023): 815–818. 36930673 10.1016/S0140-6736(23)00023-5

[27] F. Müller , J. Taubmann , L. Bucci , et al., “CD19 CAR T‐Cell Therapy in Autoimmune Disease ‐ A Case Series With Follow‐Up,” New England Journal of Medicine 390, no. 8 (2024): 687–700. 38381673 10.1056/NEJMoa2308917

[28] A. C. Pecher , L. Hensen , R. Klein , et al., “CD19‐Targeting CAR T Cells for Myositis and Interstitial Lung Disease Associated With Antisynthetase Syndrome,” JAMA 329, no. 24 (2023): 2154–2162. 37367976 10.1001/jama.2023.8753 PMC10300719

[29] J. Volkov , D. Nunez , T. Mozaffar , et al., “Case Study of CD19 CAR T Therapy in a Subject With Immune‐Mediate Necrotizing Myopathy Treated in the RESET‐Myositis Phase I/II Trial,” Molecular Therapy 32, no. 11 (2024): 3821–3828. 39245937 10.1016/j.ymthe.2024.09.009 PMC11573600

[30] C. Qin , M. H. Dong , L. Q. Zhou , et al., “Single‐Cell Analysis of Refractory Anti‐SRP Necrotizing Myopathy Treated With Anti‐BCMA CAR‐T Cell Therapy,” Proceedings of the National Academy of Sciences of the United States of America 121, no. 6 (2024): e2315990121. 38289960 10.1073/pnas.2315990121 PMC10861907

[31] R. Nicolai , P. Merli , P. Moran Alvarez , et al., “Autologous CD19‐Targeting CAR T Cells in a Patient With Refractory Juvenile Dermatomyositis,” Arthritis & Rhematology 76, no. 10 (2024): 1560–1565. 10.1002/art.42933 38924652

[32] F. Müller , A. Wirsching , M. Hagen , et al., “BCMA CAR T Cells in a Patient With Relapsing Idiopathic Inflammatory Myositis After Initial and Repeat Therapy With CD19 CAR T Cells,” Nature Medicine 31, no. 6 (2025): 1793–1797. 10.1038/s41591-025-03718-3 PMC12176613 40245922

[33] M. Hagen , F. Müller , A. Wirsching , et al., “Local Immune Effector Cell‐Associated Toxicity Syndrome in CAR T‐Cell Treated Patients With Autoimmune Disease: An Observational Study,” Lancet Rheumatology 7, no. 6 (2025): e424–e433. 40318690 10.1016/S2665-9913(25)00091-8

[34] M. Hagen , L. Bucci , S. Böltz , et al., “BCMA‐Targeted T‐Cell‐Engager Therapy for Autoimmune Disease,” New England Journal of Medicine 391, no. 9 (2024): 867–869. 39231353 10.1056/NEJMc2408786

[35] R. V. Curiel , W. Nguyen , G. Mamyrova , et al., “Improvement in Disease Activity in Refractory Juvenile Dermatomyositis Following Abatacept Therapy,” Arthritis & Rhematology 75, no. 7 (2023): 1229–1237. 10.1002/art.42450 PMC10339445 36657109

[36] Q. Tang , D. Ramsköld , O. Krystufkova , et al., “Effect of CTLA4‐Ig (Abatacept) Treatment on T Cells and B Cells in Peripheral Blood of Patients With Polymyositis and Dermatomyositis,” Scandinavian Journal of Immunology 89, no. 1 (2019): e12732. 30451307 10.1111/sji.12732

[37] H. Hasegawa , K. Kawahata , F. Mizoguchi , N. Okiyama , N. Miyasaka , and H. Kohsaka , “Direct Suppression of Autoaggressive CD8+ T Cells With CD80/86 Blockade in CD8+ T Cell‐Mediated Polymyositis Models of Mice,” Clinical and Experimental Rheumatology 35, no. 4 (2017): 593–597. 28134083

[38] A. Tjärnlund , Q. Tang , C. Wick , et al., “Abatacept in the Treatment of Adult Dermatomyositis and Polymyositis: A Randomised, Phase IIb Treatment Delayed‐Start Trial,” Annals of the Rheumatic Diseases 77, no. 1 (2018): 55–62. 28993346 10.1136/annrheumdis-2017-211751

[39] R. Aggarwal , I. E. Lundberg , Y. W. Song , A. Shaibani , V. P. Werth , and M. A. Maldonado , “Efficacy and Safety of Subcutaneous Abatacept + Standard Treatment for Active Idiopathic Inflammatory Myopathy: Phase III Randomised Controlled Trial,” Arthritis Rheumatologist 77 (2024): 765–776. 10.1002/art.43066 PMC12123255 39609094

[40] N. Xia , S. M. Hong , X. Zhang , et al., “Efficacy and Safety of Abatacept for Interstitial Lung Disease Associated With Antisynthetase Syndrome: A Case Series,” Clinical and Experimental Rheumatology 42, no. 2 (2024): 377–385. 38079347 10.55563/clinexprheumatol/53puzu

[41] S. Sukumaran and V. Vijayan , “Abatacept in the Treatment of Juvenile Dermatomyositis‐Associated Calcifications in a 16‐Year‐Old Girl,” Case Reports in Rheumatology 2020 (2020): 4073879. 32550037 10.1155/2020/4073879 PMC7275234

[42] B. Thompson , P. Corris , J. A. Miller , R. G. Cooper , J. P. Halsey , and J. D. Isaacs , “Alemtuzumab (Campath‐1H) for Treatment of Refractory Polymyositis,” Journal of Rheumatology 35, no. 10 (2008): 2080–2082. 18843768

[43] T. W. Bunch , J. W. Worthington , J. J. Combs , D. M. Ilstrup , and A. G. Engel , “Azathioprine With Prednisone for Polymyositis. A Controlled, Clinical Trial,” Annals of Internal Medicine 92, no. 3 (1980): 365–369. 6986827 10.7326/0003-4819-92-3-365

[44] A Randomized, Pilot Trial of Etanercept in Dermatomyositis,” Annals of Neurology 70, no. 3 (2011): 427–436. 21688301 10.1002/ana.22477 PMC3170432

[45] T. Nagashima and S. Minota , “Dermatomyositis in Patients With Rheumatoid Arthritis During Adalimumab Therapy,” Journal of Rheumatology 38, no. 3 (2011): 574 author reply 575. 21362792 10.3899/jrheum.100947

[46] A. Schiffenbauer , M. Garg , C. Castro , et al., “A Randomized, Double‐Blind, Placebo‐Controlled Trial of Infliximab in Refractory Polymyositis and Dermatomyositis,” Seminars in Arthritis and Rheumatism 47, no. 6 (2018): 858–864. 29174792 10.1016/j.semarthrit.2017.10.010 PMC6208161

[47] R. Campanilho‐Marques , C. T. Deakin , S. Simou , C. Papadopoulou , L. R. Wedderburn , and C. A. Pilkington , “Retrospective Analysis of Infliximab and Adalimumab Treatment in a Large Cohort of Juvenile Dermatomyositis Patients,” Arthritis Research & Therapy 22, no. 1 (2020): 79. 32293539 10.1186/s13075-020-02164-5 PMC7161150

[48] R. Shiari , M. Khalili , V. Zeinali , N. Shashaani , M. Samami , and F. H. Moghaddamemami , “Local Injection of Infliximab Into Calcinosis Lesions in Patients With Juvenile Dermatomyositis (JDM): A Clinical Trial,” Pediatric Rheumatology Online Journal 22, no. 1 (2024): 2. 38166943 10.1186/s12969-023-00941-5 PMC10759742

[49] P. Riley , L. J. McCann , S. M. Maillard , P. Woo , K. J. Murray , and C. A. Pilkington , “Effectiveness of Infliximab in the Treatment of Refractory Juvenile Dermatomyositis With Calcinosis,” Rheumatology (Oxford) 47, no. 6 (2008): 877–880. 18403404 10.1093/rheumatology/ken074

[50] M. Dastmalchi , C. Grundtman , H. Alexanderson , et al., “A High Incidence of Disease Flares in an Open Pilot Study of Infliximab in Patients With Refractory Inflammatory Myopathies,” Annals of the Rheumatic Diseases 67, no. 12 (2008): 1670–1677. 18272672 10.1136/ard.2007.077974

[51] K. S. Shaw , D. B. Reusch , R. L. Castillo , et al., “Rapid Improvement in Recalcitrant Cutaneous Juvenile Dermatomyositis With Anifrolumab Treatment,” JAMA Dermatology 160, no. 2 (2024): 237–238. 37950917 10.1001/jamadermatol.2023.4744

[52] J. H. Mulder , “Basic Cancer Chemotherapy: Experimental Models—Strategy,” Cancer Clinical Trials 1, no. 2 (1978): 129–134. 391424

[53] S. Patel , S. Jeurling , J. Albayda , E. Tiniakou , and J. Kang , “Improvement of Recalcitrant Multisystem Disease in Dermatomyositis With Anifrolumab: A Case Series,” Rheumatology (Oxford) ahead of print (2025). 10.1093/rheumatology/keaf388 PMC13396837 40663397

[54] F. Baumann Benvenuti and J. Dudler , “Long‐Lasting Improvement of Refractory Antisynthetase Syndrome With Tocilizumab: A Report of two Cases,” RMD Open 9, no. 4 (2023): e003599. 38097273 10.1136/rmdopen-2023-003599 PMC10729114

[55] Z. Lu , Y. Chen , J. Xue , and L. Liu , “NXP2‐Positive Dermatomyositis Complicated With Refractory Skin Edema: Successful Treatment With Tocilizumab,” Dermatologic Therapy 34, no. 1 (2021): e14712. 33368804 10.1111/dth.14712

[56] S. Li , W. Li , W. Jiang , et al., “The Efficacy of Tocilizumab in the Treatment of Patients With Refractory Immune‐Mediated Necrotizing Myopathies: An Open‐Label Pilot Study,” Frontiers in Pharmacology 12 (2021): 635654. 33815117 10.3389/fphar.2021.635654 PMC8010666

[57] F. Teng , J. M. Peng , Q. Wang , X. L. Tian , Z. Huo , and L. Weng , “Successful Treatment With Tocilizumab in a Patient With Rapidly Progressive Interstitial Lung Disease With Positive Anti‐Melanoma Differentiation‐Associated Gene‐5 Antibody,” Chinese Medical Journal 134, no. 8 (2020): 999–1000. 33252377 10.1097/CM9.0000000000001235 PMC8078295

[58] L. Qiu , X. Shao , L. Ma , Z. Fan , and H. Yu , “Successful Tocilizumab Treatment for Rapidly Progressive Interstitial Lung Disease With Anti‐MDA5‐Positive Juvenile Dermatomyositis: A Case Report and Literature Review,” Frontiers in Pediatrics 12 (2024): 1497168. 39664278 10.3389/fped.2024.1497168 PMC11631605

[59] X. Zhang , S. Zhou , C. Wu , et al., “Tocilizumab for Refractory Rapidly Progressive Interstitial Lung Disease Related to Anti‐MDA5‐Positive Dermatomyositis,” Rheumatology (Oxford) 60, no. 7 (2021): e227–e228. 33410494 10.1093/rheumatology/keaa906

[60] C. V. Oddis , H. E. Rockette , L. Zhu , et al., “Randomized Trial of Tocilizumab in the Treatment of Refractory Adult Polymyositis and Dermatomyositis,” ACR Open Rheumatology 4, no. 11 (2022): 983–990. 36128663 10.1002/acr2.11493 PMC9661830

[61] M. L. Kosmidis , H. Alexopoulos , A. G. Tzioufas , and M. C. Dalakas , “The Effect of Anakinra, an IL1 Receptor Antagonist, in Patients With Sporadic Inclusion Body Myositis (sIBM): A Small Pilot Study,” Journal of the Neurological Sciences 334, no. 1–2 (2013): 123–125. 23998706 10.1016/j.jns.2013.08.007

[62] T. Sugihara , N. Okiyama , N. Watanabe , N. Miyasaka , and H. Kohsaka , “Interleukin‐1 and Tumor Necrosis Factor *α* Blockade Treatment of Experimental Polymyositis in Mice,” Arthritis and Rheumatism 64, no. 8 (2012): 2655–2662. 22392010 10.1002/art.34465

[63] M. Zong , C. Dorph , M. Dastmalchi , et al., “Anakinra Treatment in Patients With Refractory Inflammatory Myopathies and Possible Predictive Response Biomarkers: A Mechanistic Study With 12 Months Follow‐Up,” Annals of the Rheumatic Diseases 73, no. 5 (2014): 913–920. 23625983 10.1136/annrheumdis-2012-202857

[64] M. Groh , K. Rogowska , O. Monsarrat , A. Denoël , P. Blanche , and L. Guillevin , “Interleukin‐1 Receptor Antagonist for Refractory Anti‐MDA5 Clinically Amyopathic Dermatomyopathy,” Clinical and Experimental Rheumatology 33, no. 6 (2015): 904–905. 26343354

[65] C. Campochiaro , N. Farina , G. de Luca , et al., “Anakinra for the Treatment of Antisynthetase Syndrome: A Monocentric Case Series and a Systematic Literature Review,” Journal of Rheumatology 50, no. 1 (2023): 151–153. 36109081 10.3899/jrheum.220213

[66] M. L. Kosmidis , D. Pikazis , P. Vlachoyiannopoulos , A. G. Tzioufas , and M. C. Dalakas , “Trial of Canakinumab, an IL‐1*β* Receptor Antagonist, in Patients With Inclusion Body Myositis,” Neurology Neuroimmunology & Neuroinflammation 6, no. 4 (2019): e581. 10.1212/NXI.0000000000000581 PMC6624107 31355317

[67] S. Julien , B. van der Woning , L. de Ceuninck , et al., “Efgartigimod Restores Muscle Function in a Humanized Mouse Model of Immune‐Mediated Necrotizing Myopathy,” Rheumatology (Oxford) 62, no. 12 (2023): 4006–4011. 37335864 10.1093/rheumatology/kead298

[68] P. Ulrichts , A. Guglietta , T. Dreier , et al., “Neonatal fc Receptor Antagonist Efgartigimod Safely and Sustainably Reduces IgGs in Humans,” Journal of Clinical Investigation 128, no. 10 (2018): 4372–4386. 30040076 10.1172/JCI97911 PMC6159959

[69] M. Yang , J. C. Yuan , Y. K. Wang , et al., “Treatment of Refractory Immune‐Mediated Necrotizing Myopathy With Efgartigimod,” Frontiers in Immunology 15 (2024): 1447182. 39502686 10.3389/fimmu.2024.1447182 PMC11534618

[70] A. Merino‐Vico , M. Kocyigit , G. Frazzei , et al., “Modulating IL‐21‐Driven B Cell Responses in Idiopathic Inflammatory Myopathies via Inhibition of the JAK/STAT Pathway,” Arthritis Research & Therapy 27, no. 1 (2025): 76. 40170058 10.1186/s13075-025-03547-2 PMC11963324

[71] M. Beckett , J. Tan , E. Bonnardeaux , et al., “Tofacitinib Therapy in Refractory Inflammatory Myositis: A Retrospective Cohort Study of 41 Patients,” Rheumatology (Oxford) 63, no. 5 (2024): 1432–1436. 37584672 10.1093/rheumatology/kead404

[72] J. J. Paik , L. Casciola‐Rosen , J. Y. Shin , et al., “Study of Tofacitinib in Refractory Dermatomyositis: An Open‐Label Pilot Study of ten Patients,” Arthritis & Rhematology 73, no. 5 (2021): 858–865. 10.1002/art.41602 PMC8084900 33258553

[73] Z. Chen , X. Wang , and S. Ye , “Tofacitinib in Amyopathic Dermatomyositis‐Associated Interstitial Lung Disease,” New England Journal of Medicine 381, no. 3 (2019): 291–293. 31314977 10.1056/NEJMc1900045

[74] J. Zhang , L. Sun , X. W. Shi , et al., “Janus Kinase Inhibitor, Tofacitinib, in Refractory Juvenile Dermatomyositis: A Retrospective Multi‐Central Study in China,” Arthritis Research & Therapy 25, no. 1 (2023): 204. 37853451 10.1186/s13075-023-03170-z PMC10583374

[75] D. Argüelles Balas , E. Barral Mena , M. Á. Martín Díaz , and E. Calvo‐Aranda , “Tofacitinib Monotherapy as Maintenance Treatment in Juvenile Dermatomyositis: A Case Report,” RMD Open 11, no. 2 (2025): e005651. 40404182 10.1136/rmdopen-2025-005651 PMC12097033

[76] Q. Zhao , Z. Zhu , Q. Fu , et al., “Baricitinib for the Treatment of Cutaneous Dermatomyositis: A Prospective, Open‐Label Study,” Journal of the American Academy of Dermatology 87, no. 6 (2022): 1374–1376. 35998841 10.1016/j.jaad.2022.08.025

[77] Z. Wang , Q. Zheng , W. Xuan , et al., “Short‐Term Effectiveness of Baricitinib in Children With Refractory and/or Severe Juvenile Dermatomyositis,” Frontiers in Pediatrics 10 (2022): 962585. 36204670 10.3389/fped.2022.962585 PMC9530147

[78] Z. Wang , Y. T. Lu , X. Qiu , et al., “Long‐Term Effectiveness and Safety of Baricitinib Treatment on Refractory or Severe Juvenile Dermatomyositis,” Rheumatology (Oxford) 64, no. 9 (2025): 4948–4956. 40316508 10.1093/rheumatology/keaf225

[79] H. Harada , H. Shoda , H. Tsuchiya , M. Misaki , T. Sawada , and K. Fujio , “Baricitinib for Anti‐Melanoma Differentiation‐Associated Protein 5 Antibody‐Positive Dermatomyositis‐Associated Interstitial Lung Disease: A Case Series and Literature Review on Janus Kinase Inhibitors for the Disease,” Rheumatology International 44, no. 5 (2024): 961–971. 38456909 10.1007/s00296-024-05551-2 PMC10980644

[80] M. Tokunaga , Y. Nakai , Y. Sato , et al., “A Pilot Transcriptomic Study of a Novel Multitargeted BRT Regimen for Anti‐MDA5 Antibody‐Positive Dermatomyositis: Improving Survival Over Conventional Therapy,” Frontiers in Immunology 16 (2025): 1568338. 40852724 10.3389/fimmu.2025.1568338 PMC12367743

[81] M. Beckett , J. Dutz , and K. Huang , “Upadacitinib Therapy in Refractory Inflammatory Myositis: A Case Series of 10 Patients,” RMD Open 10, no. 1 (2024): e003837. 38242552 10.1136/rmdopen-2023-003837 PMC10806474

[82] A. Agarwal , A. Diaz , R. al‐Dehneem , R. M. Pineda , and S. Khattri , “Off‐Label use of Janus Kinase Inhibitors in Inflammatory Cutaneous Diseases,” Journal of Drugs in Dermatology 22, no. 12 (2023): 1183–1190. 38051858 10.36849/JDD.7500

[83] T. Hornung , V. Janzen , F. J. Heidgen , D. Wolf , T. Bieber , and J. Wenzel , “Remission of Recalcitrant Dermatomyositis Treated With Ruxolitinib,” New England Journal of Medicine 371, no. 26 (2014): 2537–2538. 10.1056/NEJMc1412997 25539124

[84] S. Sengupta , B. Law , R. Sennett , J. J. Jedrych , J. Albayda , and J. K. Kang , “Ruxolitinib for Refractory PL‐12 Antisynthetase Syndrome‐Associated Angioedema‐Like Panniculitis With Clonal T‐Cell Receptor Gene Rearrangement,” JAMA Dermatology 160, no. 3 (2024): 363–366. 38117485 10.1001/jamadermatol.2023.4940

[85] L. Ladislau , X. Suárez‐Calvet , S. Toquet , et al., “JAK Inhibitor Improves Type I Interferon Induced Damage: Proof of Concept in Dermatomyositis,” Brain 141, no. 6 (2018): 1609–1621. 29741608 10.1093/brain/awy105

[86] C. Ma , M. Liu , Y. Cheng , et al., “Therapeutic Efficacy and Safety of JAK Inhibitors in Treating Polymyositis/Dermatomyositis: A Single‐Arm Systemic Meta‐Analysis,” Frontiers in Immunology 15 (2024): 1382728. 38576610 10.3389/fimmu.2024.1382728 PMC10991784

[87] T. Muchamuel , R. A. Fan , J. L. Anderl , et al., “Zetomipzomib (KZR‐616) Attenuates Lupus in Mice via Modulation of Innate and Adaptive Immune Responses,” Frontiers in Immunology 14 (2023): 1043680. 36969170 10.3389/fimmu.2023.1043680 PMC10036830

[88] M. Del Rio Oliva , C. J. Kirk , M. Groettrup , and M. Basler , “Effective Therapy of Polymyositis in Mice via Selective Inhibition of the Immunoproteasome,” European Journal of Immunology 52, no. 9 (2022): 1510–1522. 35733374 10.1002/eji.202249851

[89] S. X. Zhang , J. Wang , H. H. Sun , et al., “Circulating Regulatory T Cells Were Absolutely Decreased in Dermatomyositis/Polymyositis Patients and Restored by low‐Dose IL‐2,” Annals of the Rheumatic Diseases 80, no. 8 (2021): e130. 31611221 10.1136/annrheumdis-2019-216246

[90] M. Feng , H. Guo , C. Zhang , et al., “Absolute Reduction of Regulatory T Cells and Regulatory Effect of Short‐Term and low‐Dose IL‐2 in Polymyositis or Dermatomyositis,” International Immunopharmacology 77 (2019): 105912. 31669890 10.1016/j.intimp.2019.105912

[91] M. Miao , Y. Li , B. Huang , et al., “Treatment of Active Idiopathic Inflammatory Myopathies by low‐Dose Interleukin‐2: A Prospective Cohort Pilot Study,” Rheumatology and Therapy 8, no. 2 (2021): 835–847. 33852146 10.1007/s40744-021-00301-3 PMC8217480

[92] M. Miao , Y. Li , B. Huang , J. He , and Z. Li , “Hypomyopathic Dermatomyositis With Refractory Dermatitis Treated by low‐Dose IL‐2,” Dermatology and Therapy (Heidelb) 10, no. 5 (2020): 1181–1184. 10.1007/s13555-020-00421-8 PMC7477052 32648206

[93] Y. Zhufeng , J. Xu , M. Miao , et al., “Modification of Intestinal Microbiota Dysbiosis by low‐Dose Interleukin‐2 in Dermatomyositis: A Post hoc Analysis From a Clinical Trial Study,” Frontiers in Cellular and Infection Microbiology 12 (2022): 757099. 35360108 10.3389/fcimb.2022.757099 PMC8964112

[94] X. Zheng , R. Su , F. Hu , et al., “Low‐Dose IL‐2 Therapy Restores Imbalance Between Th17 and Regulatory T Cells in Patients With the Dermatomyositis Combined With EBV/CMV Viremia,” Autoimmunity Reviews 21, no. 11 (2022): 103186. 36084894 10.1016/j.autrev.2022.103186

[95] S. Wischnewski , H. W. Rausch , C. Ikenaga , J. Leipe , T. E. Lloyd , and L. Schirmer , “Emerging Mechanisms and Therapeutics in Inflammatory Muscle Diseases,” Trends in Pharmacological Sciences 46, no. 3 (2025): 249–263. 39939222 10.1016/j.tips.2025.01.005

[96] M.‐T. Holzer , J. F. Nies , T. Oqueka , et al., “Successful Rescue Therapy With Daratumumab in Rapidly Progressive Interstitial Lung Disease Caused by MDA5‐Positive Dermatomyositis,” Chest 163, 1 (2023): 15. 10.1016/j.chest.2022.08.2209. 36628678

[97] C.‐G. Chua , G. T. Chai , L. W. Koh , et al., “Successful Rescue Treatment of Refractory Anti‐MDA5 Autoantibody Positive Dermatomyositis with Rapidly Progressive Interstitial Lung Disease Using Daratumumab,” Clinical and Experimental Rheumatology 42, no. 2 (2024): 460–461, 10.55563/clinexprheumatol/monpb6. 38293965

[98] L. Ostendorf , U. Sester , J. M. Wintterle , et al., “Rescue Combination Treatment of Anti‐MDA5‐Associated ARDS with Daratumumab,” RMD Open 9, 3 (2023), e003238, 10.1136/rmdopen-2023-003238. 37479497 PMC10364176

[99] O. Landon‐ Cardinal , G. Guitard , F. Rigolet , et al., “Daratumumab as a Rescue Therapy in Severe Refractory Anti‐SRP Immune‐Mediated Necrotising Myopathy,” Annals of the Rheumatic Diseases 82, 4 (2023): 579–580, 10.1136/ard-2022-223541. 36609342

[100] P. Perno and P. Lisi , “Psoriasis‐Like Contact Dermatitis From a Hair Nitro dye,” Contact Dermatitis 23, no. 2 (1990): 123–124. 2209006 10.1111/j.1600-0536.1990.tb03242.x

[101] D. Chen , X. B. Wang , Y. Zhou , and X. C. Zhu , “Efficacy of Infliximab in the Treatment for Dermatomyositis With Acute Interstitial Pneumonia: A Study of Fourteen Cases and Literature Review,” Rheumatology International 33, no. 10 (2013): 2455–2458. 23715693 10.1007/s00296-012-2653-4

[102] K. A. Rouster‐Stevens , L. Ferguson , G. Morgan , C. C. Huang , and L. M. Pachman , “Pilot Study of Etanercept in Patients With Refractory Juvenile Dermatomyositis,” Arthritis Care & Research (Hoboken) 66, no. 5 (2014): 783–787. 10.1002/acr.22198 24127327

[103] A. Uruha , H. H. Goebel , and W. Stenzel , “Updates on the Immunopathology in Idiopathic Inflammatory Myopathies,” Current Rheumatology Reports 23, no. 7 (2021): 56. 34212266 10.1007/s11926-021-01017-7

[104] C. Grundtman , S. Salomonsson , C. Dorph , J. Bruton , U. Andersson , and I. E. Lundberg , “Immunolocalization of Interleukin‐1 Receptors in the Sarcolemma and Nuclei of Skeletal Muscle in Patients With Idiopathic Inflammatory Myopathies,” Arthritis and Rheumatism 56, no. 2 (2007): 674–687. 17265504 10.1002/art.22388

